# Genome-wide binding analysis unveils critical implication of B-Myb-mediated transactivation in cancers

**DOI:** 10.7150/ijbs.92607

**Published:** 2024-09-03

**Authors:** Chuntao Tao, Tao Liu, Zongrong Zhao, Xuanqi Dou, Xing Xia, Kailong Du, Xiaofeng Zuo, Yitao Wang, Tingting Wang, Youquan Bu

**Affiliations:** 1 Department of Biochemistry and Molecular Biology, College of Basic Medical Sciences, Chongqing Medical University, Chongqing 400016, China.; 2 Molecular Medicine and Cancer Research Center, Chongqing Medical University, Chongqing 400016, China.; 3 Biochemistry and Molecular Biology Laboratory, Experimental Teaching and Management Center, Chongqing Medical University, Chongqing 401331, China.

**Keywords:** B-Myb, KIF2C, Transcriptional regulation, Lung adenocarcinoma, Cancer

## Abstract

B-Myb, also known as MYB proto-oncogene like 2 (MYBL2), is an important transcription factor implicated in transcription regulation, cell cycle and tumorigenesis. However, the molecular mechanism underlying B-Myb-controlled transactivation in different cell contexts as well as its functional implication in cancers remains elusive. In this study, we have conducted a comprehensive genome-wide analysis of B-Myb binding sites in multiple immortalized or cancer cell lines and identified its critical target genes. The results revealed that B-Myb regulates a common set of core cell cycle genes and cell type-specific genes through collaboration with other important transcription factors (e.g. NFY and MuvB complex) and binding to cell type-invariant promoters and cell type-specific enhancers and super-enhancers. KIF2C, UBE2C and MYC were further validated as B-Myb target genes. Loss-of-function analysis demonstrated that KIF2C knockdown inhibited tumor cell growth both in vitro and in vivo, suppressed cell motility and cell cycle progression, accompanied with defects in microtubule organization and mitosis, strongly suggesting that KIF2C is a critical regulator of cancer cell growth and mitosis, and maintains high cancer cell motility ability and microtubule dynamics. Pan-cancer transcriptomic analysis revealed that the overexpression of both B-Myb and KIF2C presents as independent prognostic markers in various types of cancer. Notably, B-Myb associates with NFYB, binds to target gene promoters, enhancers and super-enhancers, and provokes a cascade of oncogenic gene expression profiles in cancers. Overall, our results highly suggest the critical implication of B-Myb-mediated gene regulation in cancers, and the promising therapeutic and prognostic potentials of B-Myb and KIF2C for cancer diagnosis and treatment.

## Introduction

B-Myb, also known as MYB proto-oncogene like 2 (MYBL2), is a prominent member of the MYB family of transcription factors, and is implicated in the regulation of cell cycle progression, cell proliferation, cell differentiation, cell survival, and apoptosis 1-3. It is frequently overexpressed in a broad spectrum of cancer entities, including breast cancer, non-small-cell lung cancer, colorectal cancer, neuroblastoma, osteosarcoma, etc 4-14. Overexpression of B-Myb is also significantly associated with poor prognosis in cancers, implying that B-Myb as well as its transcriptional network could be exploited as potential molecular targets for more specific anti-cancer diagnosis and therapies.

B-Myb regulates the expression of various target genes through cooperating with multiple transcriptional regulators and subsequent binding to the regulatory regions of its target genes 1. It has been showed that the canonical MYB-binding sites (MBS), cell-cycle genes homology region (CHR) elements, and FOXM1 binding motifs co-occurred in the promoters of the late cell-cycle genes 15, 16. Our previous work has highlighted the role of B-Myb and its collaboration with other transcription factors in malignant tumor development. Specifically, B-Myb accelerates colorectal cancer progression through reciprocal feed-forward transactivation of E2F2 17. Moreover, B-Myb, E2F2, and FOXM1 mutually regulate each other's expression, associate with one another, and constitute a consolidated core transcription regulatory circuity that contributes to the malignant progression of human lung adenocarcinoma (LUAD) 18. Despite extensive research advances on B-Myb-mediated transcriptional regulation, it remains not fully understood what target genes B-Myb regulates and how it regulates at genome-wide levels in different cell contexts.

In recent years, a growing body of research has underscored the significance of enhancers, particularly super-enhancers, in tumorigenesis due to their crucial role in cell growth, differentiation and cancer development 19, 20. Notably, cancer-specific super-enhancers function as principal drivers of carcinogenesis, mediating signaling pathway disorders and boosting malignant cell phenotypes. Promising therapeutic strategies have been developed to directly target super-enhancers by disrupting their structures or inhibiting their cofactors, yielding encouraging efficacy against various types of cancer 21-24.

Chromatin immunoprecipitation followed by sequencing (ChIP-seq) has revolutionized our understanding of gene regulation and epigenetic research by enabling a comprehensive analysis of DNA-binding proteins, histone modifications, and nucleosomes across the entire genome 25, 26. However, studies exploring the interactions between B-Myb and enhancers or super-enhancers are sparse. Therefore, it is of great significance to identify B-Myb cistrome at genome-wide levels using ChIP-seq to explore its functional implication in cancer development. In this study, we carried out a comprehensive analysis to systematically identify the genome-wide binding sites of B-Myb across multiple cell lines, and investigated the molecular mechanism underlying B-Myb-controlled transactivation in cancers.

## Materials and Methods

### ChIP-seq data collection and analyses

ChIP-seq data for B-Myb, H3K27ac, H3K4me1, and H3K4me3 were downloaded from the Gene Expression Omnibus (GEO) database for five different immortalized or cancer cell lines, including A673 (B-Myb: GSM3389599, H3K4me1: GSM2534114, H3K4me3: GSM2700007, H3K27ac: GSM2534350), HeLa (B-Myb: GSM665909, H3K4me1: GSM798322, H3K4me3: GSM2533939, H3K27ac: GSM733684), HepG2 (B-Myb: GSM1010876, H3K4me1: GSM798321, H3K4me3: GSM733737, H3K27ac: GSM2534179), hMEC (B-Myb: GSM1526875, H3K27ac: GSM5098095), and MCF10A (B-Myb: GSM3189834, H3K4me1: GSM3189836, H3K4me3: GSM5556561, H3K27ac: GSM5556565) 16, 27-31. Quality control assessments were conducted for all the ChIP-seq datasets using FastQC 32, 33. Low-quality reads and adapters were removed with Trim Galore. Filtered reads were mapped to the human reference genome (UCSC hg38) using Bowtie2 with default parameters 34. The SAMtools software was used for conversion of file format, removal of duplicated reads, and unique reads were retained for subsequent analysis 35.

### Prediction of Potential B-Myb Target Genes

Peak calling of each filtered B-Myb ChIP-seq dataset was performed using the MACS2 callpeak tool with default parameters 36. ChIPQC was used to calculate the percentage of read in peak (RiP%) to evaluate the signal-to-noise (SN) ratio 32. The intersectbed tool from BedTools was used to identify overlapping peaks across different cell lines 37. The annotation of overlapping peak regions to predicted potential B-Myb target genes was performed using HOMER 38.

### Enrichment analysis and PPI network construction

Gene ontology (GO) and functional pathway enrichment analyses were carried out using Panther and Reactome 39, 40. Protein-protein interaction (PPI) network was constructed using String database 41, and Cytoscape software with CytoHubba plugin was used to visualize and optimize PPI network 42, 43. Gene Set Enrichment Analysis (GSEA) was conducted to enrich the oncogenic signatures and pathways 44, 45.

### Identification of Super-Enhancers

H3K27ac ChIP-seq data were processed as described for B-Myb ChIP-seq data using MACS2 callpeak tool to identify typical enhancer regions with broad parameters. The ROSE (Ranking of Super Enhancer) algorithm was employed to identify super-enhancers in human genome reference hg38 with default parameters 46.

### Motif analysis

Motif enrichment analysis for B-Myb binding regions was conducted using HOMER de-novo algorithm with default parameters 38.

### Pan-cancer gene expression, gene correlation and survival analysis

Pan-cancer transcriptome data and clinical data were downloaded from The Cancer Genome Atlas (TCGA) data portal 47. The survival analysis for B-Myb and KIF2C in various cancers including lung adenocarcinoma was conducted by the survival and survminer packages within the R software suite. The mean value of B-Myb or KIF2C gene expression level was used as dichotomization parameter to divide the patients into high-expression and low-expression groups for survival analysis. Genes that show correlated expression with that of B-Myb in LUAD were analyzed using Pearson's correlation analysis with the corrr package. The gene expression data for B-Myb and KIF2C in pan-cancer tissues as well as its corresponding normal counterparts were download from starBase database 48, analyzed and visualized using R software.

### Cell culture, siRNAs, and transfection

Human lung adenocarcinoma cell lines A549 and H1975 were purchased from the Shanghai cell bank of the Chinese Academy of Sciences, whereas the human embryonic kidney cell line 293Ta was obtained from FulenGen (Guangzhou, China). These cells were cultured in a humidified incubator at 37°C and 5% CO2, in DMEM/F-12 (1:1), DMEM, or RPMI1640 media (Hyclone, Utah, USA) supplemented with 10% fetal bovine serum, penicillin (10^7^ U/L), and streptomycin (10 mg/L). Authentication of the cell lines was ensured through examination for mycoplasma contamination and short tandem repeat authentication. Small interfering RNAs (siRNAs) were synthesized by GenePharma (Shanghai, China) and were transiently transfected into cells using Lipofectamine RNAiMAX reagent (Invitrogen). Cells were transiently transfected with overexpression plasmids using the Lipofectamine 3000 reagent (Invitrogen).

### Stable KIF2C knockdown cell establishment

The oligonucleotides for generating KIF2C shRNA (shKIF2C) and negative control shRNA (shNC) were synthesized by Sangon (Shanghai, China), and cloned into pLKO.1-puro lentiviral vector. The sequences of the oligonucleotides were provided in Table S1. Recombinant lentiviral particles were prepared as described previously 49. Forty-eight hours after lentiviral infection, cells were selected under puromycin pressure for 72 hours to generate stable KIF2C knockdown cells.

### RNA-seq analysis

RNA-seq data for siRNA-mediated B-Myb knockdown in A549 cells were obtained from GEO database (GSE143145) 50, and reanalyzed as described previously 4. The heatmap package of R3.6.2 was used to construct heat map for visualizing gene expression.

### qRT-PCR and immunoblotting analysis

Total RNA was extracted from cells using the Trizol reagent (Invitrogen), and qRT-PCR was performed as described previously 51. For immunoblotting, cells were lysed using RIPA buffer (Beyotime, China) supplemented with protease inhibitor cocktail (Bimake, China). The total protein lysates were centrifuged and heated with loading buffer followed by SDS-PAGE analysis. The primers and antibodies used were listed in Table S1 and Table S2.

### ChIP assays and ChIP-seq

ChIP assays were conducted using the EZ-Magna ChIP™ A/G Chromatin Immunoprecipitation Kit (Millipore, Billerica, Massachusetts, USA) as described in 52, with spin column-mediated DNA purification replaced by phenol chloroform extractions to improve DNA yield and quality. Briefly, equal volumes of phenol-chloroform reagent were added to the free DNA solution eluted from magnetic beads/protein/DNA Complexes, followed by 10 seconds of vortexing and centrifugation at 13,400 g at room temperature for 8 min. The upper layer was then carefully transferred to a new EP tube, and twice the volume of pre-chilled anhydrous ethanol along with 1 μL of 2 mg/mL glycogen were added, followed by another 10 seconds of vortexing and incubation at -80°C for 30 minutes. Subsequently, the resulting pellet was obtained by centrifugation at 13,400 g at 4°C for 8 minutes, and 500 µL of cold 70% ethanol was added, followed by another round of centrifugation at 13,400 g at 4°C for 5 minutes. After air drying at room temperature for 5 minutes, 35 μL of ddH2O was added to dissolve the DNA pellet. For ChIP-seq, the VAHTS™ Universal DNA Library Prep Kit for Illumina V4 (Vazyme, Nanjing, China) was used to prepare the ChIP DNA libraries, followed by subsequent high throughput sequencing on Illumina NovaSeq Xplus platform (Illumina, CA, USA). The details of the primers and the antibodies used for ChIP were provided in Table S1 and S2, respectively.

### Cell growth, cell cycle and cell motility assays

Cell growth and cell mobility were monitored using the JULI^TM^ Stage Real-time Cell History Recorder (NanoEntek, Seoul, South Korea), and cell confluence and cell mobility were quantified as described previously 49. Cell cycle analysis was carried out as described previously 49. Wound-healing assays were conducted as previously described 51.

### EdU labeling and indirect immunofluorescence assays

EdU labeling assays were conducted using the Cell-Light EdU Apollo488 In vitro Kit (C10310-3, RiboBio, China) following the manufacturer's instructions, and indirect immunofluorescence assays were performed as described previously 18. The antibodies used were listed in Table S2.

### Tumor xenografts

A549 LUAD cells (2× 10^6^) stably infected with lentiviral particles expressing either negative control shRNA (shNC) or shRNA against KIF2C (shKIF2C) were subcutaneously injected into BALB/c-nude mice. Tumor volume was monitored and calculated as described previously 18. Thirty-four days after injection, mice were sacrificed, and tumor tissues were harvested and weighed. The animal study was reviewed and approved by Institutional Animal Care and Use Committee of Chongqing Medical University (IACUC-CQMU).

### Co-immunoprecipitation (Co-IP) assays

Co-IP assays were carried out as follows. Briefly, 293T cells were transiently co-transfected with the Flag-NFYB and B-Myb expression constructs for 48 h. Whole cell lysates were prepared and precleared with Protein A/G Magnetic Beads for 1 h at 4 °C, and then incubated with primary anti-Flag antibody (Sigma) and normal rabbit IgG (Beyotime) overnight at 4 °C. The antigen-antibody complexes were then captured by Protein A/G Magnetic Beads. Magbeaded immunoprecipitates were then separated by Magnetic separator, and finally subjected to immunoblotting analysis with the indicated antibodies.

### Protein-protein docking analysis

The three-dimensional (3D) structures of B-Myb (1-700 AA) and NFYB (1-208 AA) were predicted with high confidence (≥70) using AlphaFold v2 53. Protein-protein docking analysis was conducted using the HDOCK server, and the top three interaction models with the highest confidence score were displayed 54.

### Statistical analysis

The software package SPSS (SPSS Inc, Chicago, USA) version 21 was used to conduct routine statistical analysis. The student's t-test was applied for comparisons between two different groups. P-values below 0.05 were considered statistically significant.

## Results

### Canonical and non-canonical B-Myb binding motifs in different cellular genomes

**Figure 1:**
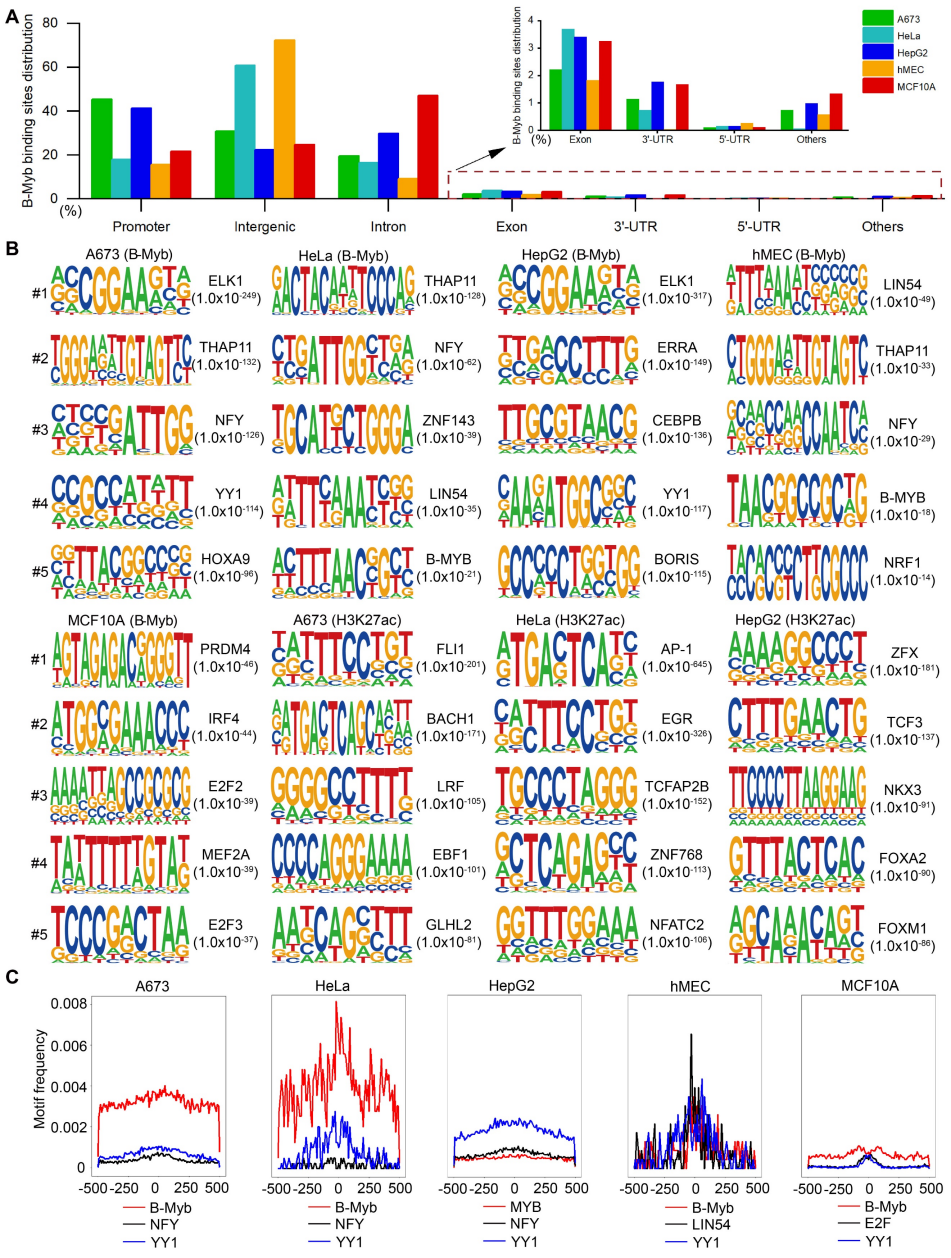
** The distribution of B-Myb binding sites and motif analysis.** (A) Genomic distributions of B-Myb binding sites. B-Myb ChIP-seq datasets for the indicated five cell lines were processed and mapped to human reference genome UCSC hg38, which is classified into promoter, intergenic, intron, exon, 5′-unstranslated region (5′-UTR), 3′-UTR, other regions (e.g. transcriptional termination site, ncRNA gene body, etc). (B) The top five motifs identified from B-Myb ChIP-seq datasets the indicated five cell lines. The H3K27ac-enriched peaks were used as controls in A673, HeLa, and HepG2 cells with available H3K27ac ChIP-seq data. Motif enrichment analysis was conducted using HOMER as described in Materials and Methods with B-Myb binding sites are shown. (C) Motif frequencies of the selected motifs. The selected motifs enriched by HOMER were plotted based on the center of B-Myb binding peaks in the indicated five cell lines.

To comprehensively study the similarities and differences of B-Myb binding sites in different cellular genomes, we analyzed B-Myb, H3K4me1, H3K4me3, and H3K27ac ChIP-seq datasets obtained in A673, HeLa, HepG2, hMEC and MCF10A cells from GEO database. All the datasets passed the quality control conducted by FastQC and ChIPQC. Trim Galore and Bowtie2 were used to filter low-quality reads and map the filtered reads to hg38 reference genome. The number of B-Myb binding sites (peaks) varied in different cell lines, with 31536, 3485, 47453, 2231 and 87704 B-Myb binding sites (peaks) identified in A673, HeLa, HepG2, hMEC, and MCF10A cell lines, respectively (Table S3). We then investigated the detailed distribution of B-Myb binding sites across the whole genome in accordance with 5'-UTR, 3'-UTR, exon, intron, intergenic, promoter, and other regions. As shown Figure 1A, majority of the B-Myb binding sites (A673: 95.8%, HeLa: 95.3%, HepG2: 93.6%, hMEC: 97.3%, and MCF10A: 93.6%) were located at intergenic, intron, and promoter regions. However, less than half of the B-Myb binding sites were located at promoter regions, highly suggesting that B-Myb not only acts on the promoter regions but also associates with distant regulatory elements, such as typical enhancers, super-enhancers, etc. Furthermore, motif enrichment analysis revealed that motifs identified in B-Myb binding sites were different among cell lines, indicating that B-Myb also collaborates with other transcription factors to regulate gene expression in different cell types. For example, ELK1, THAP11, ELK1, LIN54 and PRDM4 motifs were most significantly enriched in A673, HeLa, HepG2, hMEC and MCF10A cell lines, respectively. These motifs were specific to the B-Myb binding sites, as the motif analysis of H3K27ac data showed different motifs for the same cell line (Figure 1B). The B-Myb motif showed up in the top 5 for Hela and hMEC cells (Figure 1B), and was also significantly enriched in the other three cell lines, with ranking at 18 for A673 (P < 10^-22^), 16 for HepG2 (P < 10^-41^) and 15 for MCF10A (P < 10^-21^), respectively (data not shown). In addition, canonical B-Myb or Myb motifs as well as motifs for general transcription factors such as NF-Y and YY1 were also frequently enriched in the five cell lines (Figure 1C). Collectively, these results suggest that B-Myb regulates its target gene transcription either through recognition of the canonical binding motif or by binding to other transcription factors in different cellular contexts.

### B-Myb regulates a common set of core cell cycle genes among different cell types

**Figure 2:**
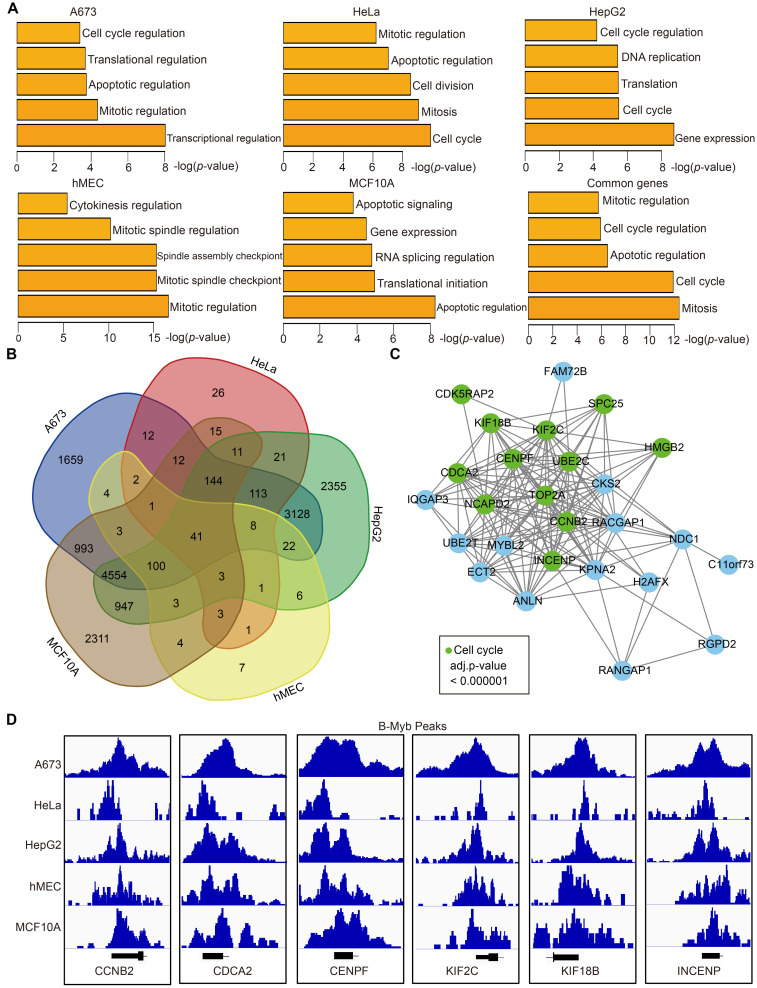
** Functional prediction of B-Myb target genes.** (A) Gene ontology analysis. The analysis was conducted with the target genes around top 500 peaks (ranked by the height of B-Myb peaks) obtained from B-Myb ChIP-seq datasets in each cell line. (B) Venn diagram of the B-Myb target gene numbers identified in the five cell lines. Forty-one B-Myb target genes are common across the five cell lines. (C) Protein-protein interaction (PPI) network. The 41 common target genes in cell cycle process were used to construct PPI network using Cytoscape. Unannotated and noncoding genes were not presented in the network. (D) The B-Myb binding peaks at the indicated B-Myb target gene promoters. The data were visualized by Integrative Genomics Viewer (IGV). Due to limited space in the figure, only the first exon for each gene was shown.

To further explore the biological function of B-Myb target genes, we annotated the genes around B-Myb binding sites using HOMER. The gene with the B-Myb binding peaks in its promoter region (±2.5kb of the transcription start site) was considered as the target gene of B-Myb. Then, gene ontology (GO) analysis was performed on the B-Myb target genes. The results revealed that the target genes around top 500 peaks in each cell line were significantly enriched in cell cycle, gene expression, and mitosis-related biological processes (Figure 2A, Table S3). The target genes of B-Myb were compared between cell lines, and 41 common genes were found among all five cell lines (Figure 2B). These 41 genes were also significantly enriched in cell cycle and mitosis-related biological processes and the expression of these genes was closely correlated with that of B-Myb (Figure S1). Protein-protein interaction (PPI)-based network analysis for these 41 common target genes was conducted to construct a core PPI network. As expected, 12/41 of these genes were related to the cell cycle (Figure 2C). Indeed, promoters of the key cell cycle-related genes (CCNB2, CDCA2, CENPF, KIF2C, KIF18B, INCENP, TOP2A, UBE2C, SPC25, HMGB2, NCAPD2, and CDK5RAP2) were strongly occupied by B-Myb in all five cell lines (Figure 2D and Figure S2). Overall, these findings highly suggest the critical function of B-Myb for controlling the expression levels of cell cycle genes in different cells.

### B-Myb regulates different genes through binding to promoters and enhancers

**Figure 3:**
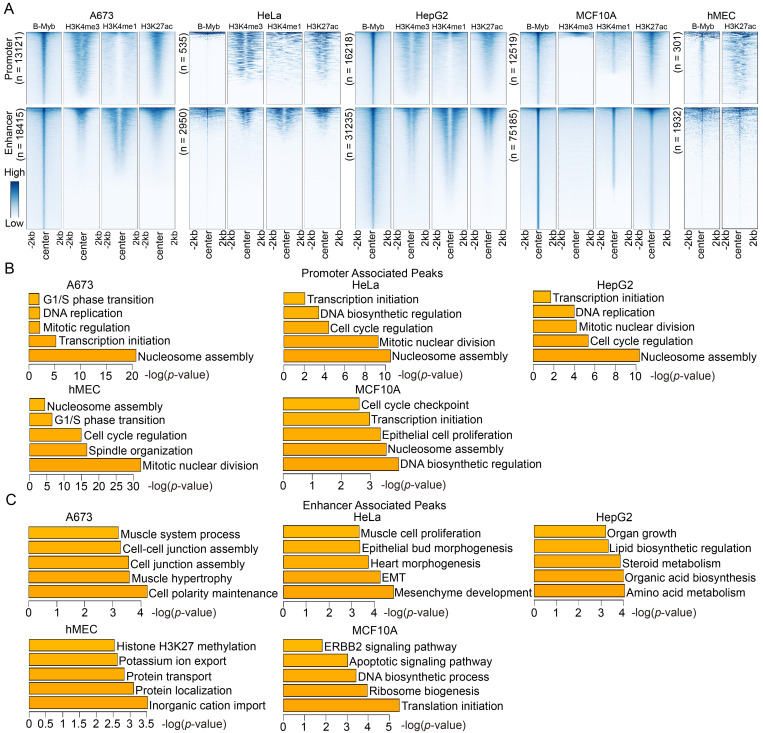
** Functional predictions of B-Myb binding peaks in promoters and enhancers.** (A) Heatmaps of B-Myb binding peaks along with active histone marks in promoter and enhancer regions. B-Myb, H3K4me3, H3K4me1 and H3K27ac ChIP-seq datasets were processed and annotated in the indicated cell lines. Each horizontal line in heatmaps represents a single binding peak for B-Myb or histone. The number of B-MYB binding sites was shown for each cell line. The y-axis was scaled to equal length. (B) Gene ontology analysis of promoter associated peaks. The top 500 target genes (ranked by the height of B-Myb peaks) obtained in each cell line were analyzed. (C) Gene ontology analysis of enhancer associated peaks. The top 500 target genes (ranked by the height of B-Myb peaks) obtained in each cell line were analyzed.

To further clarify the roles of identified B-Myb binding sites, they were categorized as either being promoter-associated (±2.5 kb of the transcription start site) or enhancer-associated, depending on their distance from the transcription start sites of known genes (Figure 3A). The results revealed that B-Myb binding sites located in enhancer regions were strongly linked to the enhancer marks (H3K4me1 and H3K27ac), indicating that B-Myb also exerts its regulatory roles through association with enhancers (Figure 3A). Although H3K4me1 and H3K4me3 ChIP-seq data are not available for hMEC cells, we combined H3K27ac ChIP-seq and TSS information to discriminate the B-Myb-bound promoter and enhancer regions in hMEC (Figure 3A). Enrichment analyses revealed that genes targeted by B-Myb through their promoter regions were predicted to be involved in mitosis-related biological processes in all five cell lines, whereas enhancer-associated B-Myb-targeted genes were found to be related to cell type-specific biological processes, such as ERBB2 signaling pathway in MCF10A cell lines (Figure 3B and 3C). These results suggest that B-Myb regulates different target genes through binding to promoters and enhancers in different cell types.

### B-Myb collaborates with LIN54/NF-Y/TEAD1 to regulate target gene transcription

**Figure 4:**
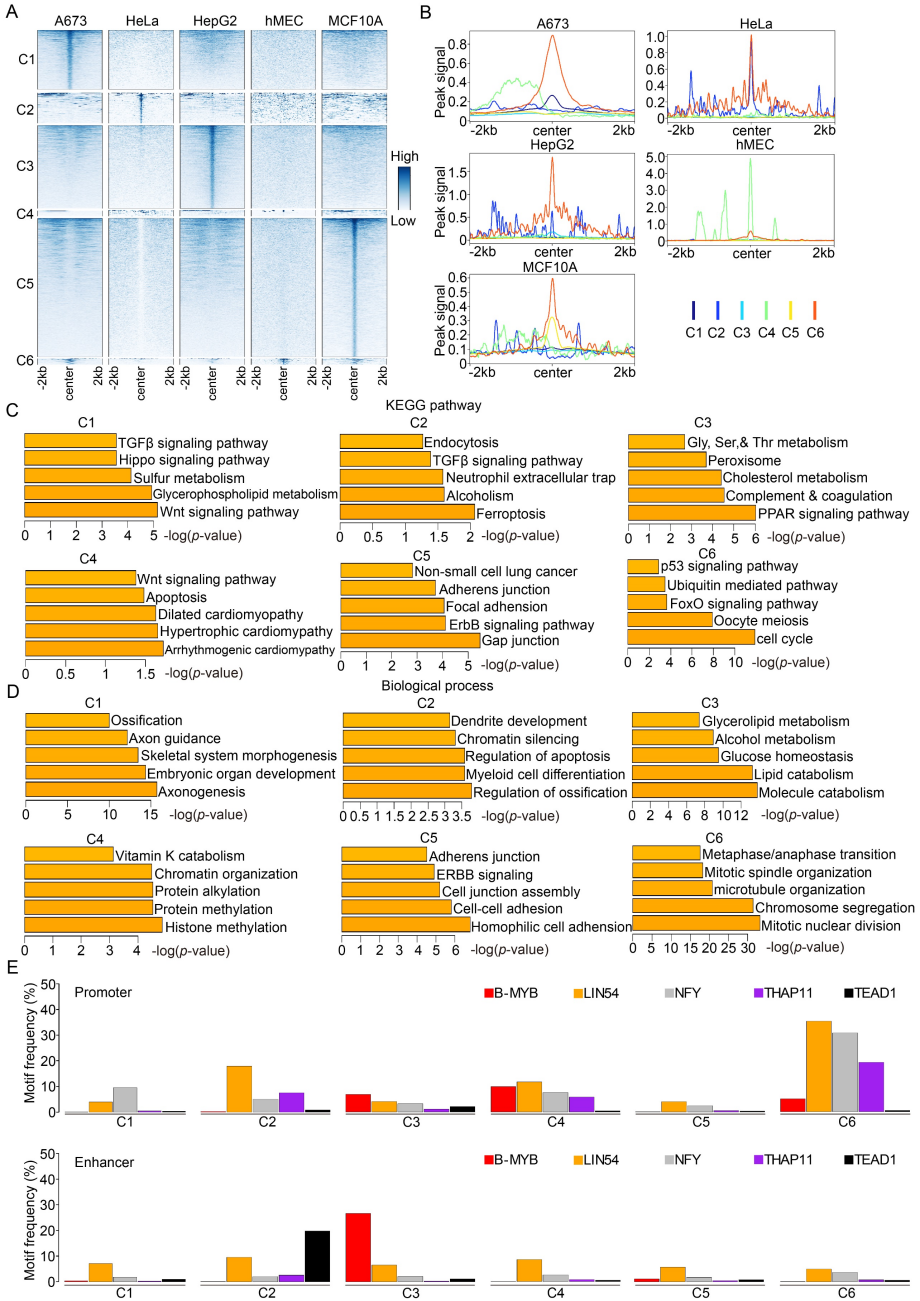
** A peak-based approach to compare B-Myb binding sites between cell lines.** (A) The heatmap of unique (C1-C5) and common (C6) clusters of B-Myb binding sites in promoter regions of the five cell lines. The intersectbed tool from BedTools was used to identify unique and common peaks across the five cell lines. (B) The line plot of B-Myb peak signals for all the C1-C6 clusters in (A). (C) KEGG pathway enrichment analysis. The top 500 genes around the B-Myb binding sites (ranked by the height of B-Myb peaks) were obtained and analyzed in each cluster. (D) Gene ontology analysis. The top 500 genes around the B-Myb binding sites (ranked by the height of B-Myb peaks) were obtained and analyzed in each cluster. (E) Motif frequency analysis. Motif enrichment analysis was conducted for each cluster by HOMER analysis. Upper panel: motif frequency for C1-C6 clusters of B-Myb binding sites in promoter regions (Fig. 4A). Lower panel: motif frequency for C1-C6 clusters of B-Myb binding sites in enhancer regions (Fig. S3). All the shown motifs are significantly enriched in all the clusters (P<0.01).

To further consolidate and extend the above findings obtained by the gene-based approach, we then turned to a peak-based approach to clearly identify the specific B-Myb binding sites (peak-centered) unique to each cell line and the common B-Myb binding sites for all cell lines (Figure 4A). The results indicated that majority of B-Myb peaks in promoter regions were unique to each cell type, while only a small fraction (n=219) of B-Myb peaks were identified in all five cell lines (marked as C6 cluster) (Table S4). In addition, the average peak density of B-Myb occupancy was enriched in all the clusters and showed the highest in the C6 cluster in four of the five cell lines (Figure 4B). GO and pathway enrichment analysis demonstrated that the cell cycle pathway was significantly associated with the C6 cluster, whereas cell type-specific pathways were found in the C1-C5 clusters, respectively (Figure 4C and 4D). For example, the C5 cluster (B-Myb peaks unique to the MCF10A cell line) was significantly associated with the ERBB signaling pathway, which has been reported to be implicated in the regulation of breast cancer progression 55. Furthermore, motif enrichment analysis demonstrated that motifs for B-Myb, LIN54, NFY, THAP11 and TEAD1 were significantly enriched in all the C1-C6 clusters, and most prominently enriched in C6 cluster, although the motif frequencies vary, highly suggesting that B-Myb regulates target genes including cell cycle genes through collaborating with LIN54, NFY, etc (Figure 4E, P<0.01). Of note, motifs for LIN54 and NFY were much highly enriched in all the clusters (Figure 4E). In consistent with our findings, B-Myb has been reported to associate with MuvB complex (LIN9, LIN37, LIN52, LIN54 and RBBP4) and TEAD1 to regulate cell cycle gene expression 56-58. NFY and THAP11 are also reported to be implicated in the regulation of cell cycle gene expression 59, 60. Moreover, similar clustering patterns of B-Myb binding peaks in enhancer regions were also observed (Figure S3 and Table S5). Motifs for B-Myb, LIN54, NFY, THAP11 and TEAD1 also significantly enriched in all the C1-C6 clusters of enhancer regions (Figure 4E). Taken together, these results suggest that B-Myb collaborates with other transcription factors such as LIN54, NF-Y and TEAD1 to regulate target gene transcription.

### B-Myb regulates cell type-specific pathways through super-enhancers

**Figure 5:**
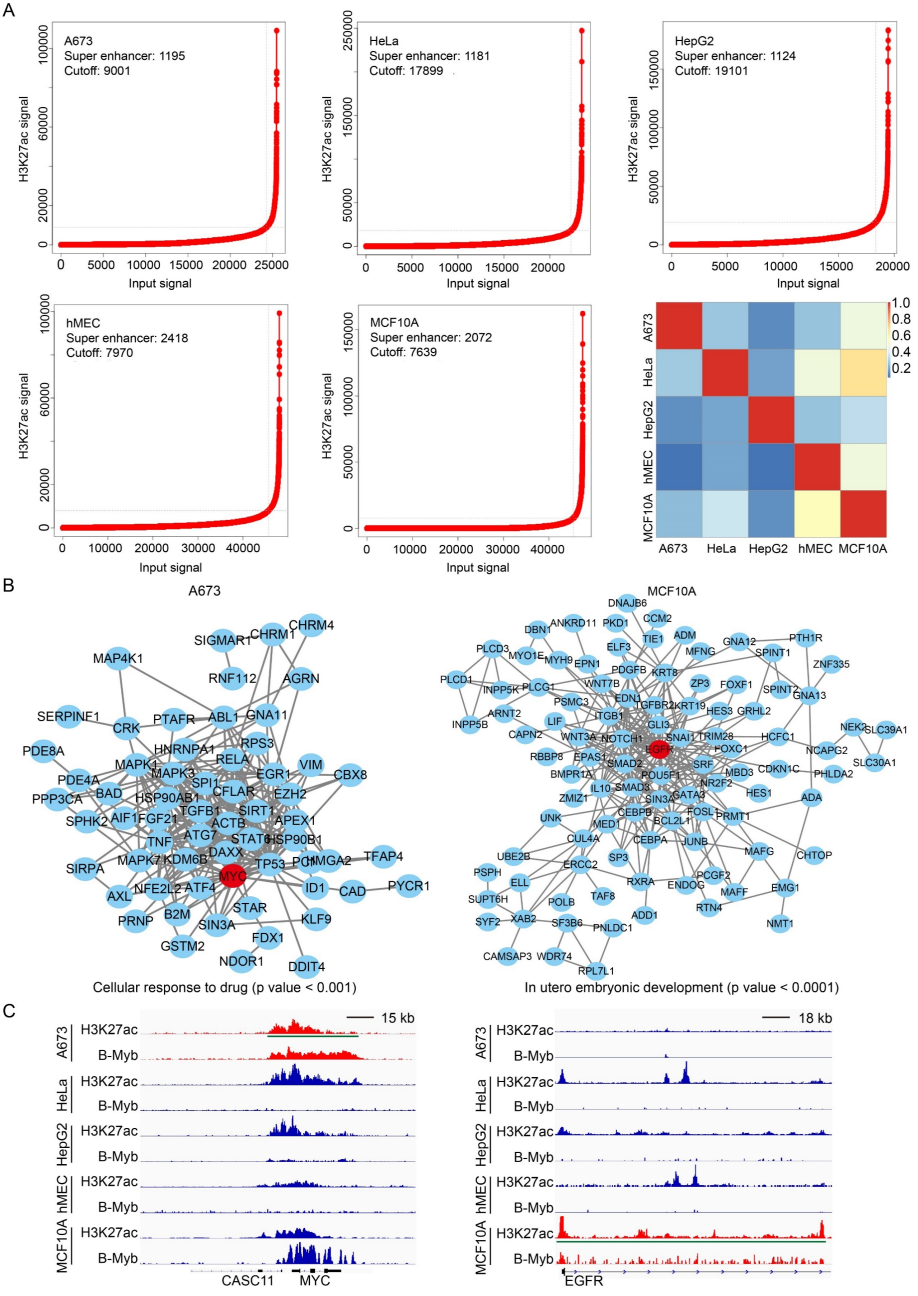
** B-Myb binding sites associated with super-enhancers.** (A) Identification of super-enhancers in the five cell lines. Super-enhancers were identified using H3K27ac ChIP-seq data with ROSE algorithm in each cell line as described in Materials and Methods. The numbers of the super-enhancers identified as well as the cutoff values are shown at each line plot. The overlap degree is indicated by different color intensities. (B) The protein-protein interaction (PPI) networks of cell type-specific biological processes in A673 and MCF10A cell lines. The core proteins, MYC (left) and EGFR (right), were highlighted in red circles. (C) Genome browser tracks at the MYC (left) and EGFR (right) gene loci illustrating the B-Myb binding and H3K27ac peaks. Due to limited space in the figure, only the first exon and intron for EGFR gene was shown. The longest super-enhancer regions for MYC in A673 and EGFR in MCF10A are indicated by the green lines, respectively. Scale bar:15kb (left) and 18kb (right).

Super-enhancer (SE) is composed of multiple constituent enhancers that regulate cell identity genes and are associated with human diseases 46. To explore the potential implication of B-Myb binding sites in super-enhancers, the super-enhancers were identified in all five cell lines using H3K27ac ChIP-seq data with ROSE algorithm. Totally, we identified 1195, 1181, 1124, 2418, and 2072 super-enhancers in A673, HeLa, HepG2, hMEC, and MCF10A cell lines, respectively (Figure 5A and Table S6). Of note, majority of the super-enhancers were exclusive to each cell line reinforcing the notion that super-enhancers are associated with cell type-specific genes. Motif analysis revealed that motifs for B-Myb, LIN54, NFY, THAP11 and TEAD1 also co-existed in B-Myb occupied super-enhancers (Figure S4). As expected, GO enrichment analysis revealed that cell type-specific biological processes could be frequently found in target genes regulated by B-Myb associated super-enhancers in each cell line (Table S7). Protein-protein interactions (PPI) networks were then constructed with the B-Myb super-enhancer-regulated genes. Of note, PPI networks with MYC and EGFR as the core proteins were identified in A673 and MCF10A cell lines, which were implicated in cell response to drug and in utero embryonic development, respectively (Figure 5B). Indeed, EGFR and MYC gene loci harbor corresponding super-enhancers which contains highly dense B-Myb binding peaks in A673 and MCF10A cell lines, respectively (Figure 5C). Overall, these results highly suggest that B-Myb regulates cell type-specific pathways through binding to the super-enhancer regions, and collaborating with transcription factors such as NFY and LIN54.

### B-Myb is overexpressed and serves as prognostic marker in cancers

**Figure 6:**
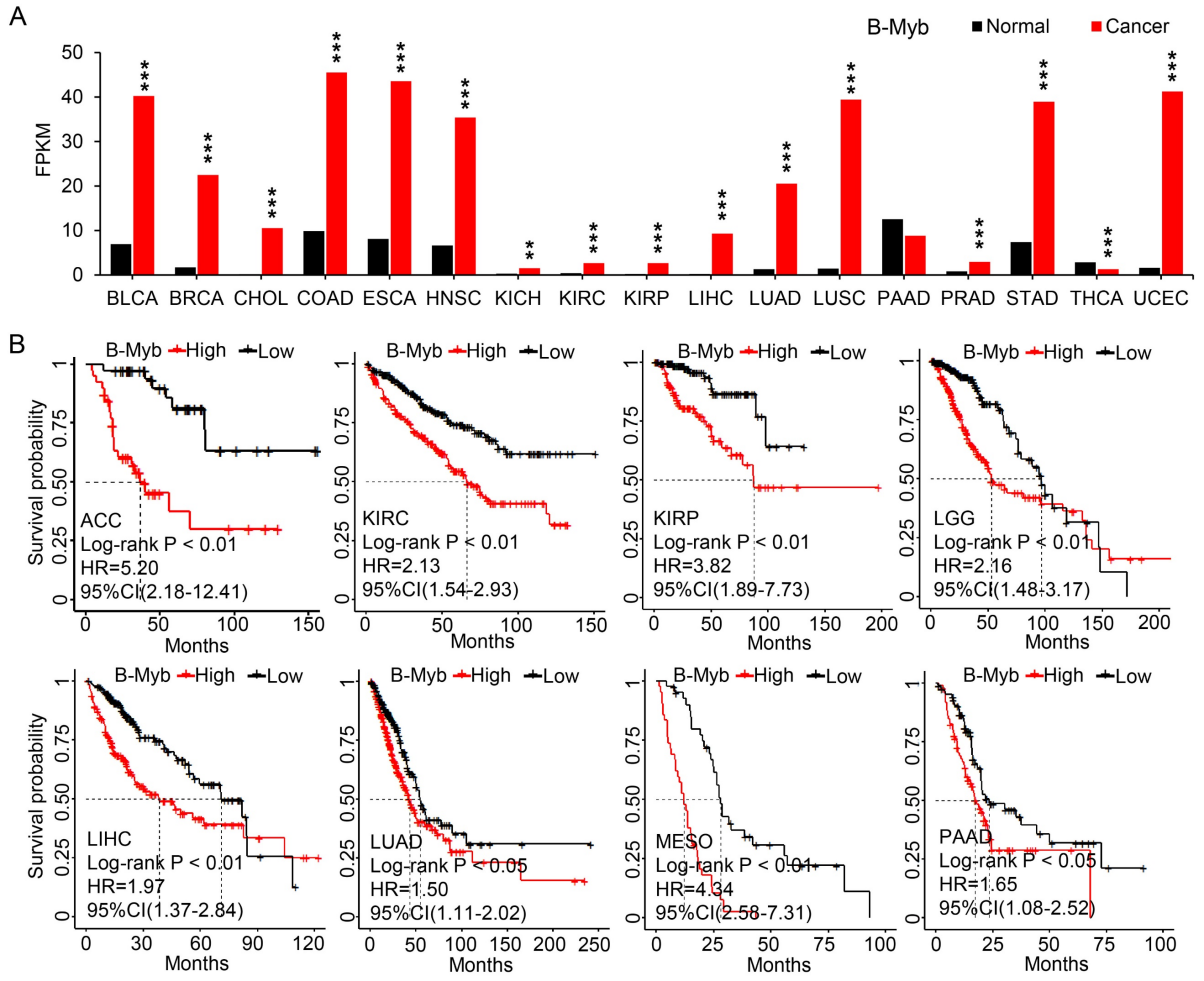
** B-Myb is overexpressed and serves as prognostic marker in cancers.** (A) B-Myb is upregulated in various types of cancers. Pan-cancer transcriptome data were downloaded from TCGA data portal and subjected to gene expression analysis. (B) B-Myb serves as prognostic marker in cancers. Probabilities for overall survival were estimated by the Kaplan-Meier method. The mean value of gene expression level was used as dichotomization parameter to divide the patients into high-expression (high) and low-expression (low) groups. ACC: Adrenocortical carcinoma; BLCA: Bladder urothelial carcinoma; BRCA: Breast invasive carcinoma; CHOL: Cholangiocarcinoma; COAD: Colon adenocarcinoma; ESCA: Esophageal carcinoma; HNSC: Head and neck squamous cell carcinoma; KICH: Kidney chromophobe; KIRC: Kidney renal clear cell carcinoma; KIRP: Kidney renal papillary cell carcinoma; LGG: Low-grade glioma; LIHC: Liver hepatocellular carcinoma; LUAD: Lung adenocarcinoma; LUSC: Lung squamous cell carcinoma; MESO: malignant mesothelioma; PAAD: Pancreatic adenocarcinoma; PRAD: Prostate adenocarcinoma; STAD: Stomach adenocarcinoma; THCA: Thyroid carcinoma; UCEC: Uterine corpus endometrial carcinoma. P<0.01 (**), P<0.001 (***).

The above genome-wide analysis of B-Myb binding sites revealed that B-Myb controls cell cycle-related genes as well as cell type-specific gene regulatory networks in cancer cells. We then systematically evaluated the expression level of B-Myb in cancers using TCGA pan-cancer transcriptomic data. The results revealed that B-Myb is remarkably upregulated across majority of the cancer cohorts in comparison to normal counterparts, including BLCA, BRCA, CHOL, COAD, ESCA, HNSC, KICH, KIRC, KIRP, LIHC, LUAD, LUSC, PRAD, STAD, THCA and UCEC (Figure 6A). The prognostic evaluation of B-Myb in TCGA cancer cohorts revealed that the expression level of B-Myb was significantly correlated with the overall survival rate of the patients with ACC, KIRC, KIRP, LGG, LIHC, LUAD, MESO, and PAAD (Figure 6B). Collectively, the results strongly suggest that B-Myb serves as a prognostic marker for various types of cancers.

### Verification of KIF2C, UBE2C, and MYC as B-Myb target genes

**Figure 7:**
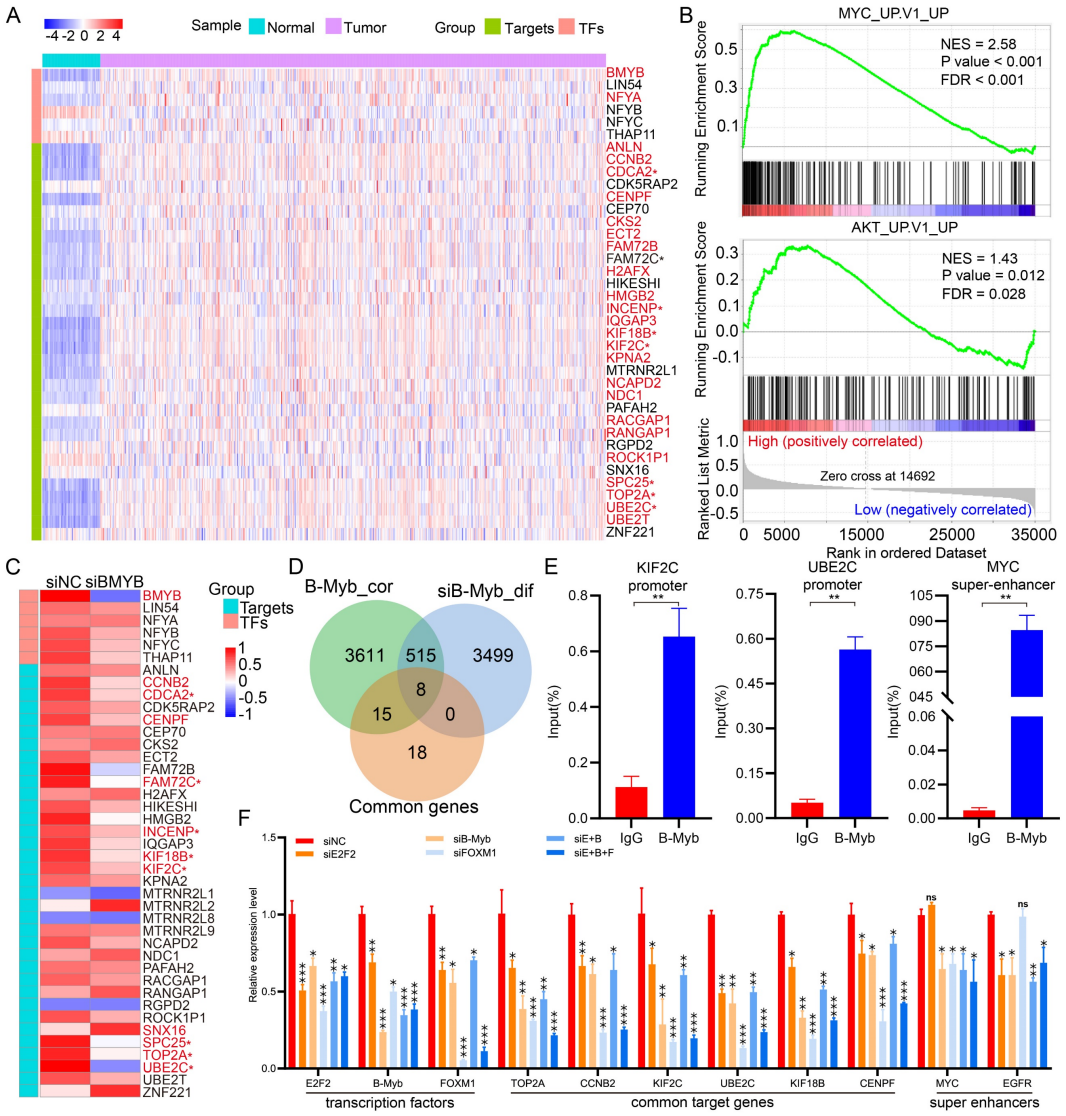
** Identification of B-Myb target genes in cancers.** (A) Heatmap of gene expression profile for the common B-Myb target genes and related transcription factors in LUAD. LUAD transcriptomic data were downloaded from TCGA data portal and analyzed. Asterisk (*) indicates the 8 overlapping genes in (D). Significantly upregulated genes are shown in red (p < 0.001, fold change ≥ 2.0). (B) GSEA plots of enriched gene sets. B-Myb-correlated genes from TCGA LUAD dataset were subjected to GSEA analysis, and representative gene sets were shown. False discovery rate, FDR. Normalized enrichment score, NES. (C) Heatmap of selected gene expression after B-Myb knockdown. B-Myb were silenced by siRNA against B-Myb, and then subjected by RNA-seq analysis in A549 cells (GSE143145). Asterisk (*) indicates the 8 overlapping genes in (D). Significantly downregulated genes are shown in red (p < 0.001, fold change ≥ 1.5). siNC: Negative control siRNA. siBMYB: B-Myb siRNA. (D) Venn diagram of overlapping genes among the indicated three groups. B-Myb_cor: highly correlated genes with B-Myb in TCGA LUAD dataset; siB-Myb_dif: differentially expressed genes upon silencing of B-Myb in A549 cells; Common genes: the 41 common target genes obtained from B-Myb ChIP-seq dataset analysis. The eight overlapping genes are marked by asterisks in (A)and (C). (E) B-Myb binds to the promoters of KIF2C, UBE2C, and MYC enhancers in vivo. The calculated data of ChIP qPCR assays performed in A549 cells with the indicated specific antibodies and control IgG are expressed as a percentage of recovered immunoprecipitated DNA relative to the Input DNA. (F) qRT-PCR verification of the gene expression after knockdown of B-Myb, E2F2, and/or FOXM1 in A549 cells. siE+B: siE2F2 + siB-Myb; siE+B+F: siE2F2 + siB-Myb + siFOXM1. The experiments were conducted at least three times independently, and the results are presented as mean and standard deviation (SD) of triplicates from a representative experiment (E and F). P<0.05 (*), P<0.01 (**), P<0.001 (***), nonsignificant (ns).

LUAD accounts for a very high cancer incidence and mortality around the world, and our latest study also highly suggested the functional implication of B-Myb in LUAD 18. Therefore, we chose LUAD as cancer model to further analyze and verify the functional implication of the 41 common B-Myb target genes in cancers. First, the expression of the set of 41 common genes (Figure 2B) along with the related transcriptional regulators (LIN54, NFYA, NFYB, NFYC and THAP11, Figure 4E) in LUAD were analyzed (Figure 7A). While eight of these 41 common target gene loci remained unannotated in current human genome database, majority of the rest well-annotated 33 common target genes were remarkably overexpressed in tumor tissues compared to their normal counterparts (Figure 7A). Then, genes with correlated expression of B-Myb in LUAD were obtained (|Pearson's correlation efficient| > 0.35 and P < 0.05). GSEA analysis revealed that B-Myb correlated genes were significantly enriched in MYC-upregulated pathway, PI3K-AKT pathway, cell cycle checkpoint process, etc (Figure 7B). Consistently, majority of the 33 common B-Myb target genes were downregulated after siRNA-mediated B-Myb knockdown in A549 cells (Figure 7C). Subsequently, through overlapping analysis on the three groups of genes, the 41 common B-Myb target genes were further narrowed down to only eight, including KIF2C, UBE2C, KIF18B, TOP2A, CDCA2, SPC25, INCENP, and FAM72C (Figure 7D). We then chose KIF2C, UBE2C and MYC for further verification. ChIP assays demonstrated that B-Myb bound to the promoter regions of KIF2C and UBE2C genes, and the enhancer regions of MYC gene in vivo (Figure 7E). We recently found that B-Myb, E2F2 and FOXM1 could constitute an exquisite core transcription regulatory circuitry that contributes to LUAD malignant progression 18. Quantitative RT-PCR analysis demonstrated that siRNA-mediated knockdown of any or all of the three transcription factors caused a decrease in the expression of KIF2C, UBE2C, KIF18B, and TOP2A, as well as the two known B-Myb target genes CCNB2 and CENPF (Figure 7F). Of note, the expression of two B-Myb-associated super-enhancer-regulated genes, MYC and EGFR, were also significantly downregulated after the knockdown of any or all the three transcription factors. These findings strongly suggest that these target genes might play a crucial role in the development and progression of LUAD. Taken together, these results clearly validate that KIF2C, UBE2C, and MYC are bona fide direct target genes of B-Myb.

### Genome-wide analysis of B-Myb binding sites in lung cancer cells

**Figure 8:**
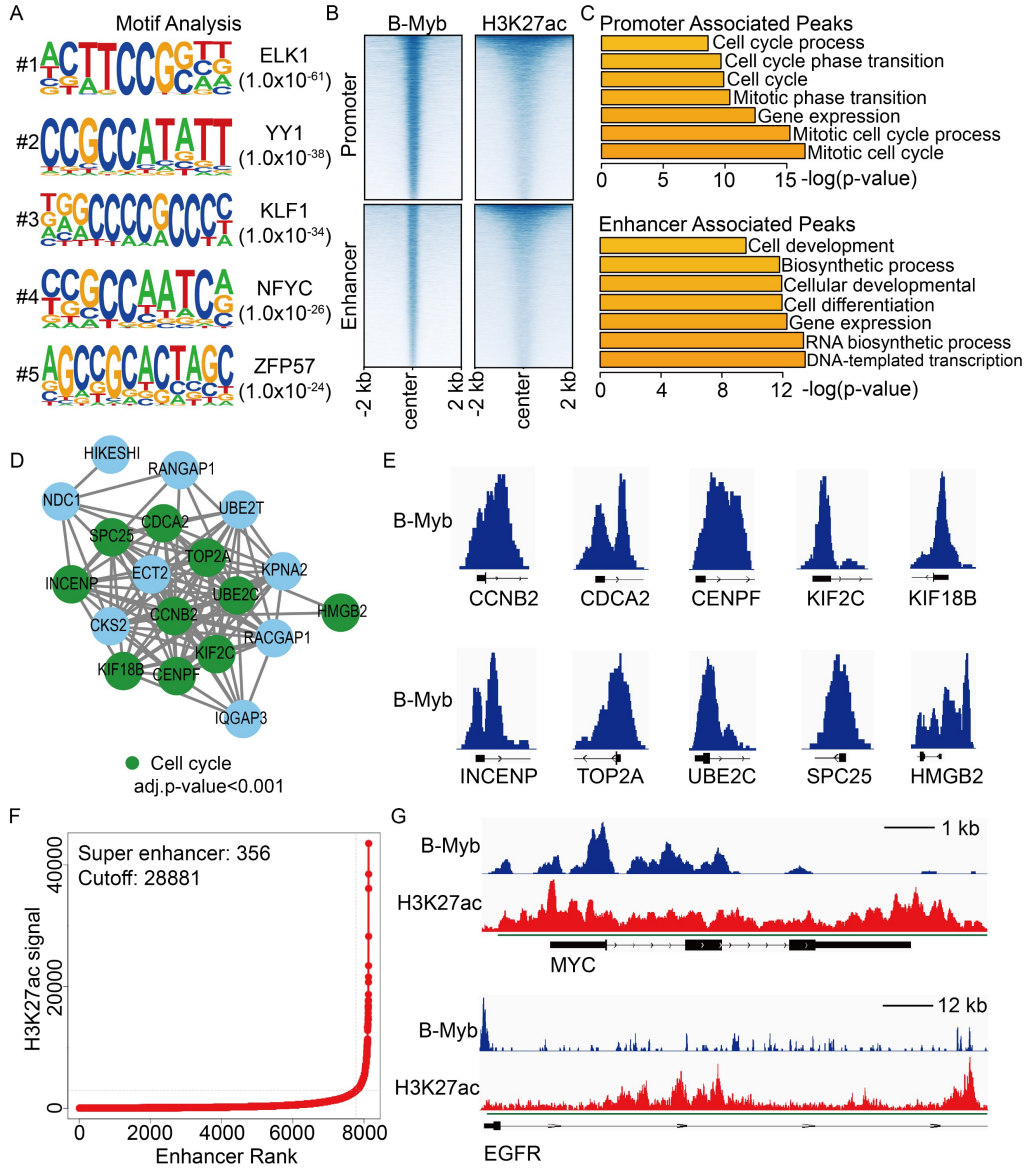
** B-Myb ChIP-seq analysis in A549 cell line.** (A) The top five motifs identified from B-Myb ChIP-seq data in A549 cell line. (B) Heatmaps of B-Myb binding peaks along with active histone marks in promoter and enhancer regions. Each horizontal line in heatmaps represents a single binding peak for B-Myb or histone. (C) Gene ontology analysis of promoter- and enhancer-associated peaks. The top 500 target genes ranked by the height of B-Myb peaks in each cell line were analyzed. (D) Protein-protein interaction (PPI) network. The 23 B-Myb target genes in A549 out of the 41 common target genes in Figure 2B were used to construct PPI network using Cytoscape. (E) The B-Myb binding peaks at the indicated target gene promoters. The data were visualized by Integrative Genomics Viewer (IGV). Only the first exon for each gene was shown. (F) Identification of super-enhancers in A549 cell line. The number of super-enhancers identified as well as the cutoff values are shown. (G) Genome browser tracks at the MYC (upper) and EGFR (lower) gene loci illustrating the B-Myb binding and H3K27ac peaks. The super-enhancer regions indicated by the green lines. Scale bar:1kb (upper) and 12kb (lower).

To further validate and make our findings more extensive, we additionally conducted ChIP-seq to analyze the genome-wide B-Myb binding in lung cancer cells A549. In total, 30683 of B-Myb binding peaks were identified (Table S8). Motif enrichment analysis revealed that motifs for ELK1, YY1, KLF1, NFY and ZFP57 were enriched at the top 5 (Figure 8A). The B-Myb motif was also significantly enriched in A549 cells with ranking at 14 (P < 10^-19^, data not shown). B-Myb binding sites located in numerous promoter and enhancer regions (Figure 8B). Enrichment analysis revealed that the target genes of the promoter-associated peak were mainly involved in cell cycle and mitosis, while enhancer-related target genes were specifically involved in cell development, differentiation, and regulation of gene expression (Figure 8C). Out of the 41 common genes found among all former five cell lines (Figure 2B), 23 common genes were identified in A549 cells (Figure 8D). Indeed, the promoter region of these genes was highly occupied by B-Myb (Figure 8E). Moreover, 356 super-enhancers were identified in A549 cells (Figure 8F), and super-enhancers in both EGFR and MYC gene loci contain highly dense B-Myb and H3K27ac binding peaks, respectively (Figure 8G). Taken together, these results highly suggest that B-Myb also regulates cell cycle-related and cell type-specific pathways in lung cancer cells, that further consolidates the findings in the former 5 cell lines.

### KIF2C is a critical regulator for cancer cell growth and mitosis

**Figure 9:**
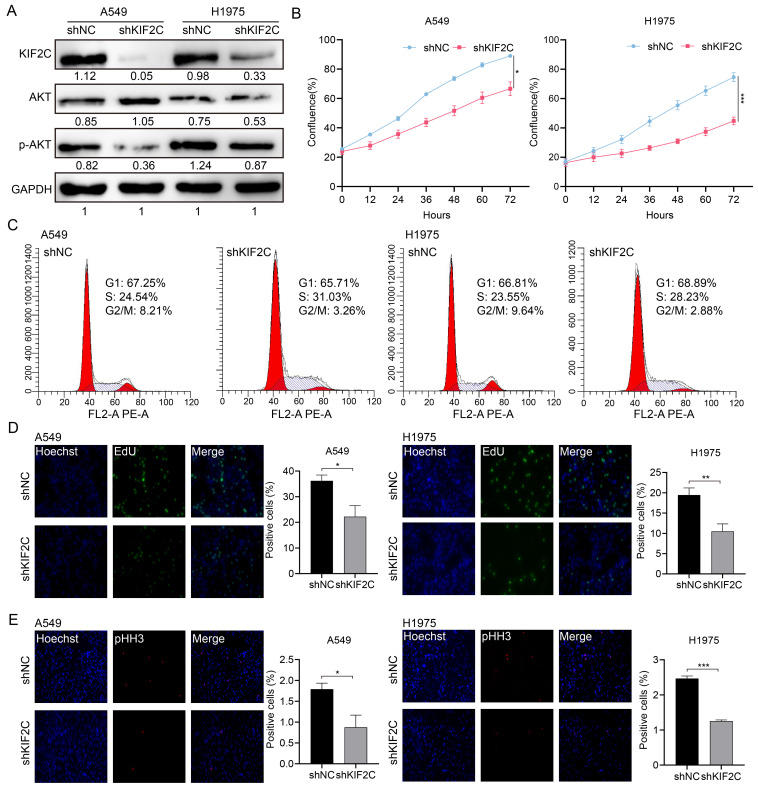
** KIF2C depletion inhibits LUAD cell proliferation and cell cycle progression.** (A) Lentivirus-mediated stable knockdown of KIF2C. Stable cells were generated by the lentivirus particles expressing negative control shRNA (shNC) and KIF2C shRNA (shKIF2C). Expression of KIF2C, AKT, and p-AKT was examined by immunoblotting analysis (B) KIF2C regulates cell proliferation. Cell growth was monitored by JULI Stage Real-time Cell History Recorder. (C) Cell cycle distribution. Cells were seeded on six-well plates, and forty-eight hours later, cells were collected and subjected to flow cytometer analysis. (D) EdU labeling. DNA biosynthesis was labeled by EdU (green), and cell nuclei was stained by Hoechst33342 (blue) in the exponentially growing KIF2C knockdown cells. The EdU positive cells were counted for statistical analysis. (E) Phospho-histone H3 (pHH3) staining. Cells were fixed and stained with anti-phospho-histone H3 (pHH3) antibody (red). The pHH3-positive cells were counted for statistical analysis. P<0.05 (*), P<0.01 (**), P<0.001 (***).

As our previous study revealed that KIF2C is a key mitotic hub gene with diagnostic and therapeutic potential in cancers such as nasopharyngeal carcinoma 49, we then selected this B-Myb target gene to examine its functional implication in the most prominent and deadly cancer type, LUAD 18. To this end, we employed two distinct LUAD cell lines, A549 and H1975, to establish stable KIF2C knockdown cells (Figure 9A). Cell proliferation assays revealed that KIF2C knockdown diminished cell growth rate in both cell lines (Figure 9B). Subsequently, flow cytometry analysis showed that KIF2C knockdown caused a pronounced delay in the progression from S phase to G2/M phase, as evidenced by the increased percentage of S phase cells and decreased percentage of G2/M phase cells in both A549 and H1975 (Figure 9C). EdU labeling analysis demonstrated that KIF2C knockdown impeded DNA biosynthesis (Figure 9D), whereas phospho-histone H3 (pHH3) staining showed that there were less pHH3-positive mitotic cells in the KIF2C knockdown group in comparison with the control group (Figure 9E). Altogether, our findings indicate that KIF2C is essential for cancer cell growth and cell cycle progression.

### KIF2C maintains high cancer cell motility ability and microtubule dynamics

**Figure 10:**
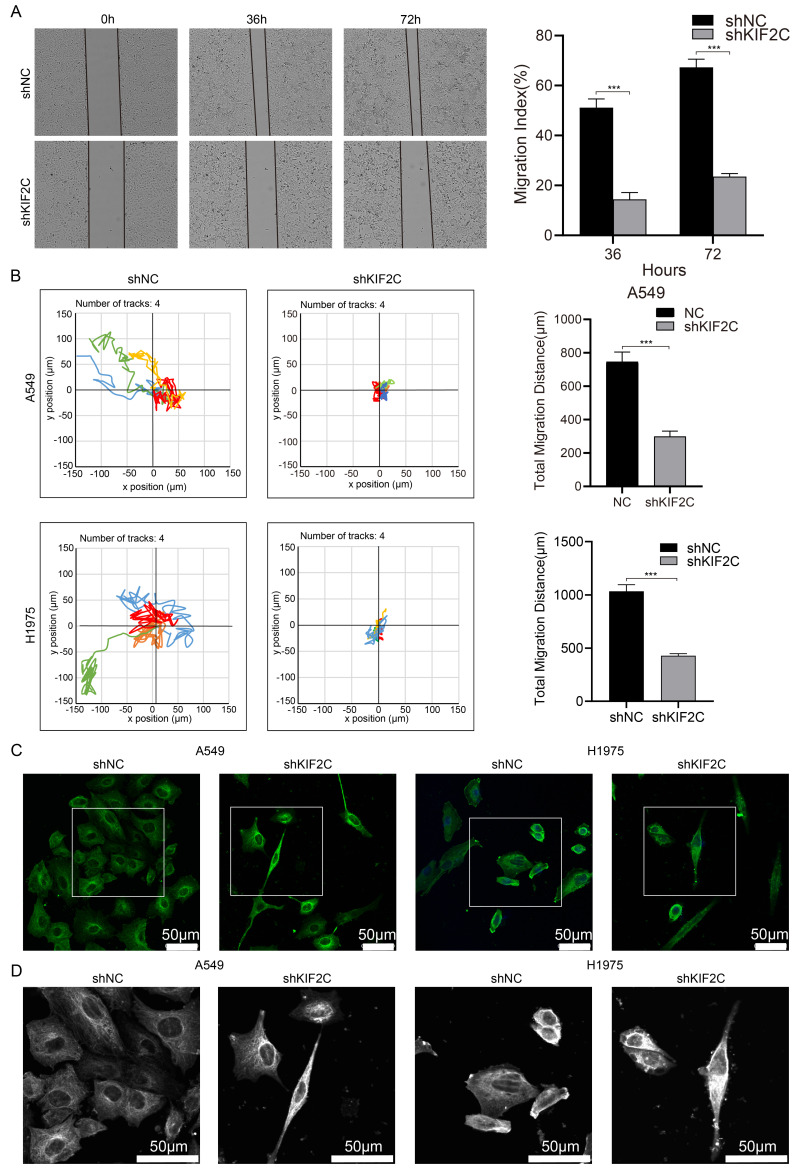
** KIF2C maintains high cancer cell motility ability and microtubule dynamics.** (A) Wound healing assays. The scratches were introduced in the stable KIF2C knockdown A549 cells, and cell migration abilities were determined at the indicated time-points. (B) Cell motility assays. The motilities of the stable KIF2C knockdown cells were monitored using JULI Stage Real-time Cell History Recorder. The motile trajectories of selected cells were presented, and the mean minutely migration speeds were quantitively calculated. (C) Immunofluorescence imaging of α-tubulin protein (green) to examine the effects of KIF2C depletion on the tubulin fiber structure in A549 and H1975 cells. Experiments were conducted in triplicate, and the data shown represent a typical experiment. (D) Zoomed original images of the boxed regions in (C). The α-tubulin proteins are shown in white for clarity. P<0.001 (***).

We then determined the effect of KIF2C on LUAD cell motility. The wound healing assays demonstrated that KIF2C knockdown resulted in a remarkably decreased rate of wound closure in both cell lines (Figure 10A). These findings were further substantiated by the real-time cell motility assessments conducted using a live cell imaging system, which illustrated that KIF2C knockdown led to a lower overall distance and mean velocity of A549 and H1975 cells in comparison to the control groups (Figure 10B). Moreover, immunofluorescence staining of α-tubulin revealed that KIF2C knockdown caused significant aggregation of tubulin fibers, as well as a noticeable decrease in size and entanglement of tubulin fibers in comparison to the regular extended, orderly structure observed in control cells (Figure 10C and 10D), thereby strongly indicating that KIF2C has a considerable role in regulating microtubule dynamics and thus maintaining high cancer cell migration and motility abilities.

### Therapeutic and diagnostic values of KIF2C in cancers

**Figure 11:**
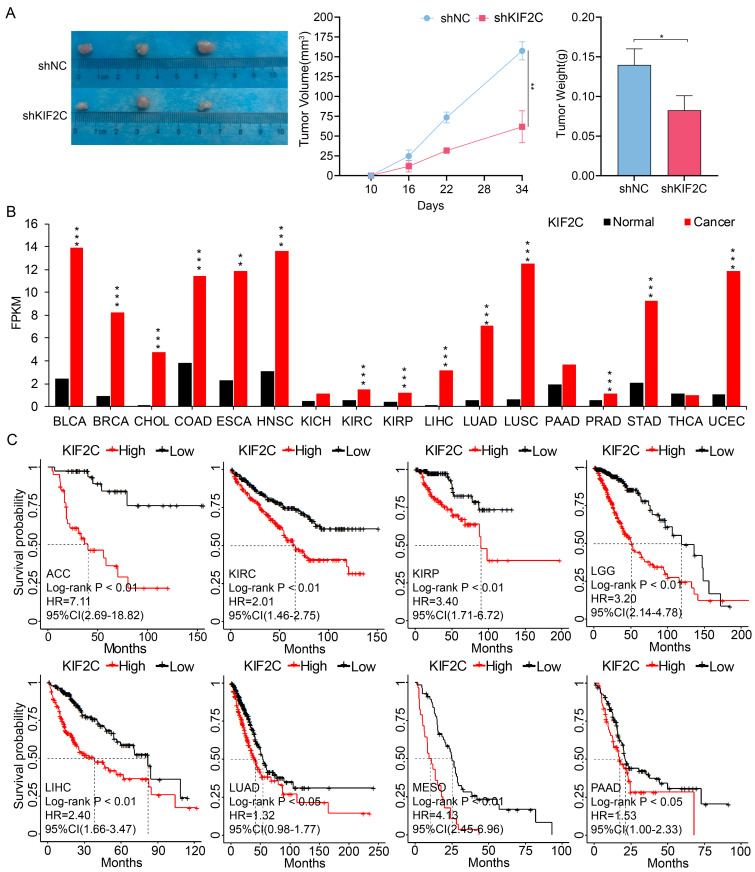
** Therapeutic and prognostic values of KIF2C in cancers.** (A) KIF2C is required for LUAD growth in vivo. A549 cells with stable KIF2C knockdown as well as the control cells were injected subcutaneously into the dorsal flanks of nude mice. The tumors were monitored regularly for 5 weeks and excised at the end of the experiment. shNC: negative control shRNA; shKIF2C: KIF2C shRNA. (B) KIF2C is upregulated in various types of cancers. Pan-cancer transcriptome data were downloaded from TCGA data portal. (C) KIF2C presents as prognostic marker in cancers. Probabilities for overall survival were estimated by the Kaplan-Meier method. The mean value of KIF2C gene expression was used to divide the patients into high-expression (high) and low-expression (low) groups. P<0.05 (*), P<0.01 (**), P<0.001 (***).

Finally, we aimed to evaluate the therapeutic and diagnostic values of KIF2C in cancers. We demonstrated the effects of KIF2C on LUAD in vivo, by observing a considerable decrease in tumor growth following KIF2C knockdown in nude mice compared to the control group, which suggests that KIF2C is a critical gene for tumor growth in vivo in LUAD (Figure 11A). We then systematically compared the expression level of KIF2C between normal and tumor tissues using TCGA pan-cancer transcriptomic data. The results revealed that KIF2C is remarkably upregulated across majority of the pan-cancer cohorts in comparison to normal tissues, including BLCA, BRCA, CHOL, COAD, ESCA, HNSC, KIRC, KIRP, LIHC, LUAD, LUSC, PRAD, STAD and UCEC (Figure 11B). The prognostic evaluation of KIF2C in TCGA cancer cohorts revealed that the expression level of KIF2C was significantly correlated with the overall survival rate of the patients with ACC, KIRC, KIRP, LGG, LIHC, LUAD, MESO, and PAAD (Figure 11C). Overall, the results highly suggest that KIF2C serves as a promising therapeutic target for various types of cancers including LUAD.

### B-Myb associates with NFYB

**Figure 12:**
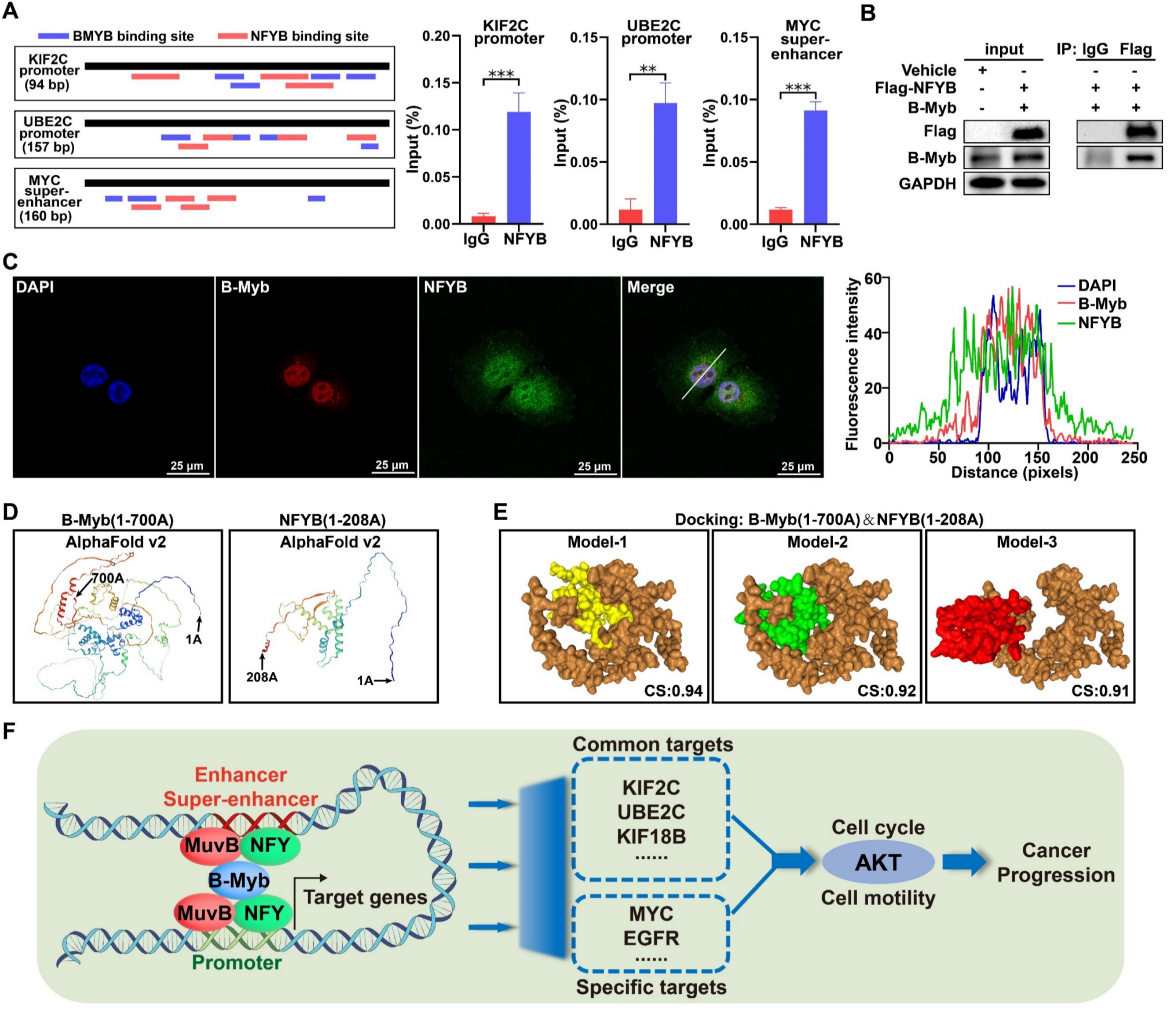
** B-Myb interacts with NFYB.** (A) NFYB binds to KIF2C and UBE2C promoters, and MYC super-enhancers in vivo. The consensus binding sites for B-Myb and NF-Y are illustrated in the indicated regulatory regions amplified by ChIP qPCR (left panel). The calculated data of ChIP qPCR assays performed in A549 cells with specific NFYB antibody and control IgG are expressed as a percentage of recovered immunoprecipitated DNA relative to input DNA (right panel). (B) B-Myb associates with NFYB in vivo. 293T cells were transiently transfected with the indicated expression plasmids for B-Myb and NFYB for 48 hours, and then cell lysates were prepared and subjected to co-immunoprecipitation assays. (C) B-Myb colocalizes with NFYB in cell nuclei. H1299 cells were transfected with B-Myb and Flag-NFYB expression plasmids for 48 hours, and then cells were fixed and stained with anti-B-Myb antibody (red) and anti-Flag antibody (green), and intensity spatial profiles were plotted. Scale bar = 25 μm. (D) Three-dimensional (3D) structures of B-Myb and NFYB. The 3D structures of B-Myb (1-700 AA) and NFYB (1-208 AA) were predicted by AlphaFold v2 online. (E) Predicted docking models of B-Myb and NFYB interaction. The homologous docking analysis for B-Myb and NFYB interaction was conducted using HDOCK online server, and the top three models with the highest confidence score were displayed. B-Myb is shown in brown, and NFYB are shown in yellow, green and red, respectively. CS: Confidence score. (F) Mechanistic working model. B-Myb regulates a common set of target genes and cell type-specific genes through collaboration with other transcription factors (e.g. NFY and MuvB complex) and binding to cell type-invariant promoters and enhancers/super-enhancers, and subsequently activates AKT pathways and promotes malignant progression.

The aforementioned data suggest that B-Myb might collaborate with other transcription factors especially NFY and LIN54 to bind to promoters, typical enhancers and super-enhancers. As the interaction between B-Myb and LIN54 has been reported 56, we then determined to investigate the potential interaction between B-Myb and NFYB, which is one prominent subunit of heterotrimeric NF-Y. Transcription factor binding analysis revealed that KIF2C and UBE2C gene promoters as well as MYC super-enhancer contain multiple consensus binding motifs for both B-Myb and NFYB (Figure 12A). Consistent with the observation in Figure 7E, ChIP assays further revealed that NFYB also bound to KIF2C and UBE2C gene promoter and MYC super-enhancer in vivo (Figure 12A). Co-immunoprecipitation assay with NFYB-tagged antibody confirmed that anti-Flag-NFYB immunoprecipitates contained B-Myb, suggesting that B-Myb forms a complex with NFYB in vivo (Figure 12B). Immunofluorescence assays showed that B-Myb colocalized with NFYB in cell nuclei (Figure 12C). We further conducted a molecular docking analysis for B-Myb and NFYB through the HDOCK server. Three-dimensional (3D) structures of B-Myb(1-700 AA) and NFYB(1-208 AA) were predicted by AlphaFold v2 (Figure 12D). The detailed molecular docking analysis revealed three top-scored homologous docking models for interaction between B-Myb and NFYB (Figure 12E). Collectively, our findings indicate that B-Myb regulates a common set of target genes and cell type-specific genes through collaboration with other transcription factors (e.g. NFY and MuvB complex) and binding to cell type-invariant promoters and cell type-specific enhancers and super-enhancers, and subsequently activates oncogenic pathways such as PI3K-AKT and promotes malignant progression in cancers.

## Discussion

### B-Myb-mediated gene regulation is critically implicated in cancers

B-Myb, a highly conserved transcription factor belonging to the Myb family, plays a crucial role in regulating the expression of target genes and associated signaling pathways via binding to the respective promoter regions and recruiting additional transcription factors. Previous studies on B-Myb mainly focused on its ability to activate the expression of late S-phase and late G2-phase genes through binding to the MuvB complex and recruiting FOXM1, respectively 16, thereby regulating cell cycle progression. However, very limited studies have explored the implication of B-Myb in cancer cell motility and metastasis. Our previous study revealed that overexpression of B-Myb upregulated downstream gene expression and activated PI3K-AKT signaling pathway, which are associated with cancer metastasis 17. Our present findings revealed that B-Myb also transactivated target genes implicated in the biological processes such as epithelial-mesenchymal transition (EMT), adherens junction, focal adhesion, etc. Therefore, our study extends our current understanding of B-Myb target genes to the functional involvement in cell metastasis.

Previous studies have suggested that the B-Myb-MuvB complex regulates the expression of genes in the G2/M phase of the cell cycle only by binding to the promoter region of genes. Pattschull *et al* have subsequently found that B-Myb can also regulate the expression of G2/M phase genes by indirectly binding to the enhancer region of target genes through interaction with YAP 31. Another recent study on the B-Myb-MuvB complex have found that the pioneer transcription factor complex formed by B-Myb and MuvB can bind directly to nucleosomes, and regulate chromatin accessibility 61. In addition, other studies have showed that NF-Y contains histone-fold domain (HFD), and regulates not only housekeeping genes through cell type-invariant promoter binding, but also cell identity genes by binding to cell type-specific enhancers to facilitate permissive chromatin conformations 62-64. Of note, our motif analysis indeed demonstrated that B-Myb binding peaks enriched in binding motifs for both B-Myb and NF-Y in both promoter and enhancer regions. Our results further proved that B-Myb and NF-Y associated with each other, and bound to promoters and enhancers of target genes (e.g. KIF2C, UBE2C, MYC). Our data also indicated that the B-Myb binding peaks remarkably varied in all the five cell lines in this study, highly suggesting that B-Myb and NF-Y are implicated in regulating the cancer cell identity and plasticity. Moreover, in MCF10A cells, data revealed that majority of the B-Myb-binding promoters are not actively transactivated (Figure 3A), suggesting the potential role of B-Myb in the transcriptional repression of target genes. Our lab is currently focusing on lung cancer cell lines to deeply investigate the role of B-Myb and NF-Y in lung cancer cell plasticity and development.

Our previous study revealed that B-Myb not only forms a reciprocal feed-forward transactivation loop with E2F2, but also collaborates with FOXM1 and E2F2 to constitute a consolidated core transcription regulatory circuity, which enhances the activation of target genes. This gene transcription regulatory network formed by multiple transcription factors is of great significance for maintaining cell homeostasis and promoting malignant tumor progression 18. We therefore propose that B-Myb might collaborate with NF-Y, FOXM1, E2F2 and even other transcriptional regulator to constitute a more elaborated transcription regulatory circuitry implicated in cancer cell plasticity and cancer development, which is of broad physio-pathological significances and warrants deep studies in future.

### B-Myb and KIF2C are promising diagnostic and therapeutic targets for cancers

Previous studies have reported that B-Myb is overexpressed and associated with poor prognosis in a variety of cancers including breast cancer, non-small cell lung cancer, colorectal cancer, neuroblastoma, osteosarcoma, esophageal cancer, and multiple myeloma 4-11. In this study, our results further demonstrated the prognostic value of B-Myb expression in pan-cancers such as adrenocortical carcinoma, renal clear cell carcinoma, renal papillary cell carcinoma, low-grade glioma of the brain, hepatocellular carcinoma, lung adenocarcinoma, mesothelioma, and pancreatic cancer, highly suggesting the necessity for deep investigations on the role of B-Myb in these cancers and the underlying molecular mechanisms.

As a typical transcription factor, B-Myb is known to regulate a variety of target genes such as CCNB1 and CDK1 in cell cycle regulation, BCL2 and BIRC5 in cell survival, SOX2 in cell differentiation, and SNAI1 in cell invasion 1. Previous studies reported that B-Myb regulates the expression of canonical oncogene MYC via binding to its promoter region, and B-Myb-MuvB complex could regulate the transcription of mouse UBE2C gene via binding to its CHR elements in promoter region 65, 66. KIF2C and UBE2C have been also suggested to be potential target genes of B-Myb 3. Here our study further revealed that B-Myb also transactivates MYC transcription through binding to the super-enhancer of MYC, and verified that KIF2C and UBE2C are bona fide target genes of B-Myb.

Aberrant KIF2C expression has been observed in cancers and overexpression of KIF2C is associated with cancer progression, invasion, metastasis, and poor prognosis, particularly in breast 67, 68, gastric 69, and colorectal cancer 70, 71. Our recent work identified KIF2C as a pivotal regulator of cell cycle progression and cell motility in nasopharyngeal carcinoma 49. Although one previous bioinformatic analysis reported that KIF2C associates with LUAD progression and prognosis 72, its functional implication in LUAD remains largely elusive. In this study, our functional analysis revealed that KIF2C knockdown remarkably repressed LUAD cell proliferation, cell cycle progression and cell motility. In vivo xenograft nude mouse models verified that KIF2C was a critical gene for tumor growth in LUAD. Overall, our study strongly suggests that B-Myb and its critical target gene KIF2C are promising diagnostic and therapeutic targets for cancers including LUAD.

## Conclusions

In summary, our research conducted a comprehensive analysis of the genome-wide binding sites of B-Myb and identified its key target genes across distinct cell lines. B-Myb regulates a common set of cell cycle genes as well as cell-type-specific genes through binding to their promoters, enhancers, or super enhancers. KIF2C, UBE2C, and MYC are three bona fide target genes regulated directly by B-Myb. KIF2C is a critical regulator of cancer cell growth and mitosis, and maintains high cancer cell motility ability and microtubule dynamics. B-Myb associates with NFYB, and thus provokes a cascade of oncogenic gene expression profiles in cancers. Our results highly suggest the critical implication of B-Myb-mediated gene regulation in cancers, and the promising therapeutic and prognostic potentials of B-Myb and KIF2C for cancer diagnosis and treatment.

## Supplementary Material

Supplementary figures and tables.
