# Supplementary material for: Genome-wide binding analysis unveils critical implication of B-Myb-mediated transactivation in cancers
Source: Int J Biol Sci. 2024 Sep 3;20(12):4691–712. doi: 10.7150/ijbs.92607 (PMC11414393; doi:10.7150/ijbs.92607)
Supplement: Supplementary file 1 — Supplementary figures and tables. [file ijbsv20p4691s1.pdf]

Supplementary Figures

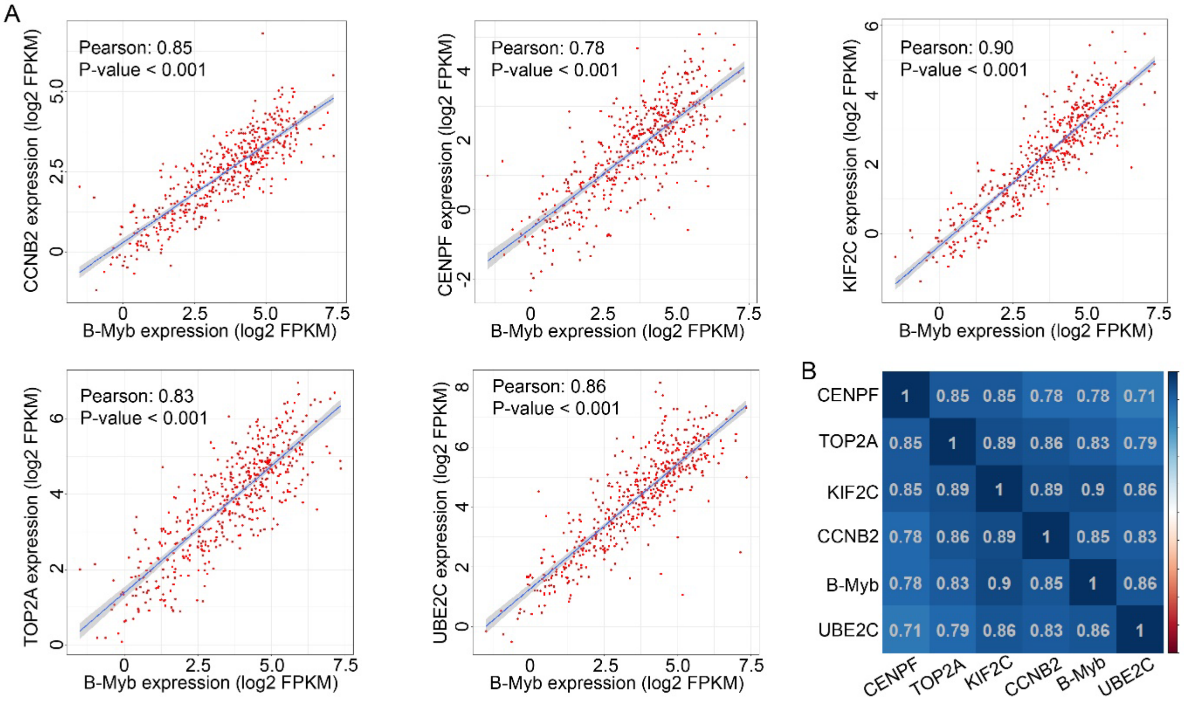

Figure S1. Positive expression correlation between B-Myb and its target genes. (**A**) Pearson correlation analysis of B-Myb and its highly correlated target genes in LUAD samples. TCGA LUAD transcriptome data were downloaded and subjected to gene correlation analysis. Normalized expression (log2(FPKM)) levels of the indicated genes in LUAD tissues (red dot) were plotted as scatter plots, and Pearsons' correlation coefficients (Pearson) were calculated; (**B**) Heatmap of Pearson correlation coefficient between B-Myb and its highly correlated target genes as shown in (A).

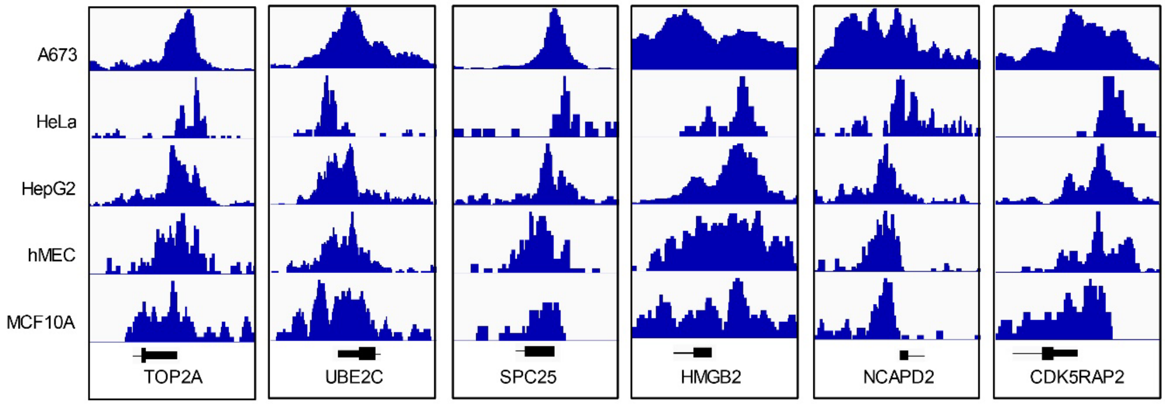

Figure S2. B-Myb binding peaks in its target gene promoters. The B-Myb binding peaks at the indicated B-Myb target gene promoters. The data were visualized by Integrative Genomics Viewer (IGV). Due to limited space in the figure, only the first exon for each gene was shown.

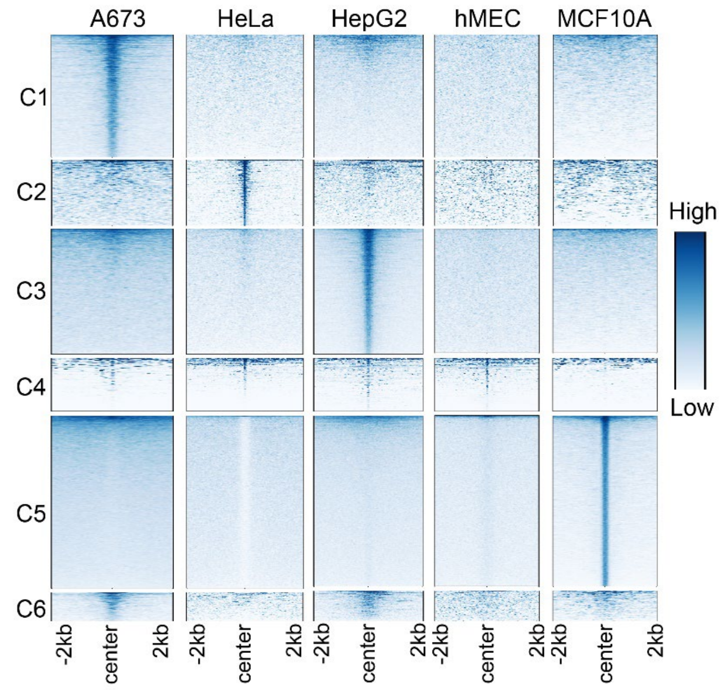

Figure S3. Heatmap of unique and common clusters of B-Myb binding sites in enhancer regions.

The intersectbed tool from BedTools was used to identify unique (C1-C5) and common (C6) peaks across the five cell lines. C1: A673; C2: HeLa; C3: HepG2; C4: hMEC; C5: MCF10A; C6: common.

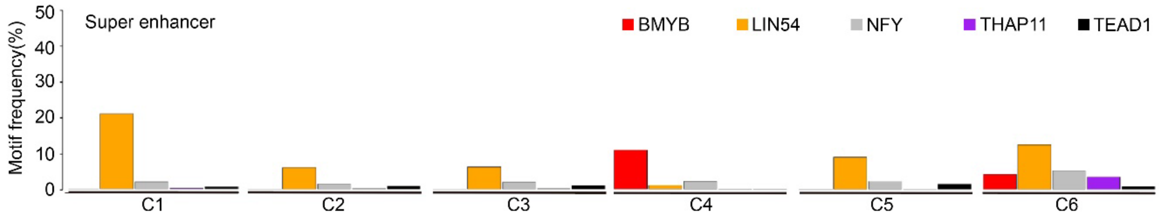

Figure S4. Motif frequency analysis for B-Myb associated super-enhancers. Super-enhancers identified in Fig 5A were used to identify unique (C1-C5) and common (C6) clusters across the five cell lines. C1: A673; C2: HeLa; C3: HepG2; C4: hMEC; C5: MCF10A; C6: common. Motif enrichment analysis was conducted for each cluster by HOMER analysis. All the shown motifs are significantly enriched in all the clusters (P<0.01).

Supplementary Tables

Table S1. shRNAs and Primers used in this study.

The shRNA sequences.

Gene name	Primer name and sequences
Negative control shRNA (NCsh)	Sense: 5'-*ACCGGT*TGGTTTACATGTTGTGTGACTCGAG TCACACAACATGTAAACCATTTTT-3'<br>Antisense: 5'- *GAATTC*AAAAATGGTTTACATGTTGTGTGA CTCGAG TCACACAACATGTAAACCA-3'
KIF2C shRNA (KIF2Csh)	Sense: 5'-*CCGG*CGCCCACTGAATAAGCAAGAACTCGAG TTCTTGCTTATTCAGTGGGCGTTTTT-3'<br>Antisense: 5'- *AATT*AAAAACGCCCACTGAATAAGCAAGAA CTCGAGTTCTTGCTTATTCAGTGGGCG-3'

Primers used for qRT-PCR analyses

Gene name	Primer name and sequences
GAPDH	F833: ACCTGACCTGCCGTCTAGAA<br>R1060: TCCACCACCCTGTTGCTGTA
E2F2	F809: ACTCGGTATGACACTTCGCT<br>R1036: TCTGGTGGGGTCTTCAAACA
KIF18B	F13655: TACGAGGACACGTACAACACC<br>R14675: CAGGCTGGTCACATTGCTC
UBE2C	F1785: TCAGACAACCTTTTCAAATGGGT<br>R2938: AGCGAGAGCTTATACCTCAGG
MYC	F238: TACAACACCCGAGCAAGGAC<br>R2121: TTCTCCTCCTCGTCGCAGTA
DEPDC1	F1911: CGCAGCCCTCTATGCTATTCA<br>R2052: AGTGGCGAGTCTCGGCACAA
KIF2C	F945: CTACAGGTTCACAGCAAGGC<br>R1148: TTCCGGTAGCAGGGTTGATT
EGFR	F135041: CCCACTCATGCTCTACAACCC<br>R136913: TCGCACTTCTTACACTTGCGG
B-Myb	F484: AAGATGTTGCCAGGGAGGAC<br>R679: TGGTCAGAAGACTTCCCTGG
FOXM1	F1071: ATACGTGGATTGAGGACCACT<br>R1245: TCCAATGTCAAGTAGCGGTTG
CENPF	F1198: CTCTCCCGTCAACAGCGTTC<br>R1299: GTTGTGCATATTCTTGGCTTGC

Primers used for ChIP-PCR analyses

Primer name	sequences(5 to 3')
KIF2C-F1979	ACAGCGGAATGACGTTTTAAG
KIF2C-R2178	GCGTTGGATCTTGATAGCG
UBE2C-F1795	GCCACCAATTCGCTACGGAT
UBE2C-R1951	TGATCCAGCCAATGAGACGC
MYC-F1768	AGGGTGATGTTCATTAGCAGTGG
MYC-R1927	AACAAGTTTCCAGCCACCTCC

Table S2. Antibodies used in the present study.

Protein name	Manufacturer (cat. number)	Applications (working dilution)	Website Link
GAPDH	Xianzhi Bio (AB-P-R 001)	IB (1:5000)	http://www.goodhere.com/showproduct.asp?id=320&classid=34&nid=2
KIF2C	Abcam (ab187652)	IB (1:1000)	https://www.abcam.cn/products/primary-antibodies/mcak-antibody-epr14838-ab187652.html
AKT	Selleck (A5031)	IB (1:1000)	https://www.selleck.cn/antibodies/akt1-2-3-rabbit-recombinant-mab.html
α-tubulin	CST (2144)	IF (1:100)	https://www.cellsignal.cn/products/primary-antibodies/a-tubulin-antibody/2144
p-AKT	Selleck (A5030)	IB (1:1000)	https://www.selleck.cn/antibodies/phospho-akt-ser473-rabbit-recombinant-mab.html
p-HH3	CST (53348)	IF (1:100)	https://www.cellsignal.cn/products/primary-antibodies/phospho-histone-h3-ser10-d7n8e-xp-rabbit-mab/53348
IgG	CST (2729)	IP (1:50)	https://www.cellsignal.cn/products/primary-antibodies/normal-rabbit-igg/2729
Flag	SIGMA (F1804)	IB (1:1000)	https://www.sigmaaldrich.com/GB/en/product/sigma/f1804
B-Myb	Abcam (ab191064)	IB (1:1000)	https://www.abcam.cn/products/primary-antibodies/b-myb-antibody-n-terminal-ab191064.html
B-Myb	Abcam (ab191064)	IF (1:100)	https://www.abcam.cn/products/primary-antibodies/b-myb-antibody-n-terminal-ab191064.html
Flag	SIGMA (F1804)	IF (1:100)	https://www.sigmaaldrich.com/GB/en/product/sigma/f1804

Table S3 to Table S8

The file sizes of Table S3 to Table S8 are too large to be uploaded and shown here. However, these supplementary tables are available from the corresponding author upon reasonable request.
